# Identifying chondroprotective diet-derived bioactives and investigating their synergism

**DOI:** 10.1038/s41598-018-35455-8

**Published:** 2018-11-21

**Authors:** Rose K. Davidson, Jonathan Green, Sarah Gardner, Yongping Bao, Aedin Cassidy, Ian M. Clark

**Affiliations:** 1 Biomedical Research Centre, School of Biological Sciences, University of East Anglia, Norfolk, NR4 7TJ UK; 2 Bob Champion Research and Education Building, Norwich Medical School, Department of Nutrition, Norwich Research Park, University of East Anglia, Norfolk, NR4 7UQ UK

## Abstract

Osteoarthritis (OA) is a multifactorial disease and nutrition is a modifiable factor that may contribute to disease onset or progression. A detailed understanding of mechanisms through which diet-derived bioactive molecules function and interact in OA is needed. We profiled 96 diet-derived, mainly plant-based bioactives using an *in vitro* model in chondrocytes, selecting four candidates for further study. We aimed to determine synergistic interactions between bioactives that affected the expression of key genes in OA. Selected bioactives, sulforaphane, apigenin, isoliquiritigenin and luteolin, inhibited one or more interleukin-1-induced metalloproteinases implicated in OA (*MMP1*, *MMP13*, *ADAMTS4*, *ADAMTS5*). Isoliquiritigenin and luteolin showed reactive oxygen species scavenging activity in chondrocytes whereas sulforaphane had no effect and apigenin showed only a weak trend. Sulforaphane inhibited the IL-1/NFκB and Wnt3a/TCF/Lef pathways and increased TGFβ/Smad2/3 and BMP6/Smad1/5/8 signalling. Apigenin showed potent inhibition of the IL-1/NFκB and TGFβ/Smad2/3 pathways, whereas luteolin showed only weak inhibition of the IL-1/NFκB pathway. All four bioactives inhibited cytokine-induced aggrecan loss from cartilage tissue explants. The combination of sulforaphane and isoliquiritigenin was synergistic for inhibiting *MMP13* gene expression in chondrocytes. We conclude that dietary-derived bioactives may be important modulators of cartilage homeostasis and synergistic relationships between bioactives may have an anti-inflammatory and chondroprotective role.

## Introduction

Osteoarthritis (OA) is a global problem and a leading cause of disability^1,2^. OA is the most common type of arthritis affecting 13.2% of males and 19.8% of females in the UK^3^. There are no effective disease-modifying drugs for OA, with pharmacological treatment restricted to pain relief. Treatment of chronic conditions such as osteoarthritis (OA) is further complicated by co-morbidities as the risk escalates with age^4^. The importance of the health, economic and societal issues surrounding OA was acknowledged by the General Assembly of the United Nations and World Health Organisation^5,6^.

OA is a disease affecting the entire di-arthrodial joint where degeneration of the articular cartilage, changes in the underlying (subchondral) bone and synovial inflammation are typically seen. This disease is a major cause of disability, that results in a loss of mobility in the affected joint and unmanaged pain symptoms are detrimental to quality of life^7^. Metalloproteinases commonly linked to the destruction of articular cartilage are the collagenase MMP-13 and aggrecanases ADAMTS-4 and ADAMTS-5^8,9^.

A change in the typical approach to chronic disease management may benefit patients^10^. The consequence for conditions of slow progression such as OA is a late presentation of symptoms, when joint damage has already occurred. This may in part account for the lack of effective disease-modifying interventions for OA. With age as a primary risk factor for OA, the entire population can be considered as ‘at risk’ for OA disease. Dietary intervention as a strategy to reduce risk at the population level provides a low risk option with potentially wide-reaching benefits^11^.

Available epidemiological data suggest that dietary constituents, specifically carotenoids, n3-polyunsaturated fatty acids (PUFA), vitamin K and allium intake are associated with a reduction in the progression of OA in man^12^. A number of other plant-derived phytochemicals outside of nutrients themselves, have been proposed to have benefits on joint health and OA e.g.^13,14^ but translation to man has been slow. Detailed molecular studies both *in vitro* and *in vivo* are required in order to establish modes of action, which are different from traditional single target pharmaceuticals, and to optimise clinical trial design in order to gain optimal evidence of efficacy in both disease treatment and prevention.

Previously, our research in this area has focused on sulforaphane (SFN), an isothiocyanate derived from the glucosinolate precursor glucoraphanin, which is abundant in cruciferous vegetables, particularly broccoli. It is widely known for its cytoprotective, anti-inflammatory, anti-microbial and anti-tumourigenic activities. There are a number of reports on the potential for SFN in OA describing protection of chondrocytes from cell death, repression of catabolic metalloproteases, intra-articular administration using microspheres to protect cartilage, protection of subchondral bone and cartilage protection in mouse models of OA^15–19^.

We sought to extend knowledge in this area by screening a library of diet-derived bioactives for inhibition of either basal or interleukin-1-induced expression of matrix metalloproteinase-13 (*MMP13*) in two chondrocyte-like cell lines as a surrogate for chondroprotection. The top ‘hits’ from this screen were further tested in primary human articular chondrocytes which led us to focus on four bioactives for possible synergism (sulforaphane (SFN), apigenin (API), isoliquiritigenin (ISO) and luteolin (LUT)). These compounds were then examined for mechanism of action but also their interaction.

Flavonoids are polyphenolic compounds common to a wide range of plant foods. Flavones are a flavonoid sub-class to which apigenin and luteolin belong. Apigenin is found abundantly in bell peppers, garlic, belimbi fruit, guava, celery, parsley and chamomile. Sources of luteolin are bird’s eye chilli, onions, carrots and olive oil^20,21^. Luteolin is reported to inhibit aggrecanases ADAMTS4 and ADAMTS5 in ATDC5 cells and murine cartilage explants^22^ and apigenin is a hyaluronidase inhibitor as demonstrated by *in vitro* turbidity assay and in bovine cartilage explants^23,24^, and is reported to inhibit metalloproteinase expression in primary rat articular chondrocytes and the human SW1353 chondrosarcoma cell line^25,26^.

Isoliquiritigenin is also a flavonoid found in liquorice root, though has a chalcone structure. Chalcone derivatives are shown to have wide ranging bioactivities such as anti-inflammatory, anti-oxidant, anti-microbial activity^27^. Isoliquiritigenin is reported to protect subchondral bone in a mouse model of OA^28^, repress interleukin-1-induced matrix metalloproteinases in rat primary chondrocytes^29^, protect cartilage in the anterior cruciate ligament transection (ACLT) mouse model of OA, and inhibit the inflammatory nuclear factor kappa B (NFκB) pathway in a rat model of intracerebral haemorrhage and ATDC5 cells^30,31^, also affecting the NLRP3 inflammasome and inducing NRF2 signalling^30^.

## Results

### Key matrix metalloproteinases in osteoarthritis: diet-derived bioactives inhibit gene expression

In order to identify compounds which were potentially chondroprotective, ninety six diet-derived bioactives were screened at 10 μM against basal and IL-1-induced *MMP13* expression (measured by RT-qPCR) in SW1353 human chondrosarcoma and C28/I2 immortalised human costal chondrocytes. Each screen was performed once *n* = 1, with three replicates of each condition, with the 10 μM dose chosen pragmatically in order to reveal activity.

The heat-map (Supplementary data S1) shows distinct areas of high and low *MMP13* expression for IL-1 treated SW1353 cells. Two clades (highlighted) of low *MMP13* expression across the two cell lines were noted to contain apigenin, isoliquiritigenin and luteolin.

From these initial screens, ten bioactives were selected and with sulforaphane (identified in our previous research^16^) they were tested for inhibition of *MMP13* expression, but also other cartilage matrix-degrading proteases *MMP1*, *ADAMTS4* and *ADAMTS5*. Human primary articular chondrocytes (HACs) isolated from three separate patients *n* = 3, were treated with 10 µM genistin, luteolin, polydatin, curcumin, apigenin, myricetin, isoliquiritigenin, ursolic acid, epigallocatechin gallate, naringin or sulforaphane in triplicate for 6 hours, or pre-treated with 10 µM of each bioactive for 30 mins then 5 ng/ml IL-1 for 6 hours. Levels of protease gene expression are shown in Table 1 (IL-1-induced) and Table 2 (basal).

**Table 1:** Inhibition of cytokine-induced levels of key osteoarthritis matrix metalloproteinase mRNA expression in primary chondrocytes.

Induced	MMP1		MMP13		ADAMTS4		ADAMTS5	
Bioactive	Mean FC (95% CI)	p-value	Mean FC (95% CI)	p-value	Mean FC (95% CI)	p-value	Mean FC (95% CI)	p-value
Genistin	0.75 (−0.88 to 1.39)	0.994	0.89 (−0.62 to 0.84)	0.999	0.81 (−0.50 to 0.88)	0.997	0.77 (−0.62 to 1.07)	0.996
**Luteolin**	0.30 (−0.43 to 1.84)	0.429	0.39 (−0.12 to 1.34)	0.201	0.41 (−0.10 to 1.28)	0.068	0.27 (−0.11 to 1.58)	0.075
Polydatin	0.73 (−0.86 to 1.41)	0.997	0.74 (−0.47 to 0.99)	0.987	0.80 (−0.49 to 0.89)	0.997	0.84 (−0.68 to 1.01)	0.999
Curcumin	0.61 (−0.74 to 1.53)	0.899	0.62 (−0.35 to 1.11)	0.799	0.83 (−0.52 to 0.86)	0.992	0.44 (−0.28 to 1.41)	0.124
**Apigenin**	0.23 (−0.36 to 1.91)	0.186	0.18 (0.09 to 1.55)	*0*.*005*	0.11 (0.20 to 1.58)	*0*.*0001*	0.22 (−0.06 to 1.62)	*0*.*018*
Myricetin	0.97 (−1.10 to 1.16)	0.999	1.07 (−0.80 to 0.66)	0.999	1.34 (−1.03 to 0.35)	0.994	0.77 (−0.61 to 1.07)	0.991
**Isoliqu**	0.23 (−0.36 to 1.90)	0.151	0.14 (0.14 to 1.59)	*0*.*0005*	0.08 (0.15 to 1.69)	*0*.*0001*	0.11 (0.05 to 1.73)	*0*.*001*
Ursolic acid	0.82 (−0.95 to 1.32)	0.991	0.31 (−0.03 to 1.43)	*0*.*041*	0.69 (−0.38 to 1.00)	0.902	0.33 (−0.17 to 1.52)	0.136
EGCG	1.03 (−1.16 to 1.11)	0.999	1.01 (−0.74 to 0.72)	0.999	1.27 (−0.95 to 0.42)	0.999	0.97 (−0.81 to 0.88)	0.999
Naringin	0.94 (−1.07 to 1.20)	0.999	1.13 (−0.86 to 0.60)	0.999	1.16 (−0.85 to 0.53)	0.999	0.97 (−0.81 to 0.87)	0.999
**Sulforaphane**	0.17 (−0.31 to 1.96)	0.163	0.12 (0.15 to 1.61)	*0*.*001*	0.03 (0.28 to 1.66)	*0*.*0001*	0.23 (−0.07 to 1.61)	0.053

Cytokine-induced matrix metalloproteinase gene expression in human articular primary chondrocytes pre-treated with 10 µM of each bioactive 30 mins then 5 ng/ml interleukin-1 (IL-1) for 6 hours. Cell isolates from three patients (*n* = 3) were used and each independent experiment performed in triplicate. Levels of *MMP1*, *MMP13*, *ADAMTS4* and *ADAMTS5* mRNA, normalised to *18S* were measured by RT-qPCR. Mean fold change (FC) to IL-1, 95% confidence intervals (CI) are shown. One-way ANOVA, and all treatments were compared to IL-1 treatment with Dunnett’s post-test corrected p-values shown.

**Table 2:** Inhibition of basal levels of key osteoarthritis matrix metalloproteinase mRNA expression in primary chondrocytes.

Basal	MMP1		MMP13		ADAMTS4		ADAMTS5	
Bioactive	Mean FC (95% CI)	p-value	Mean FC (95% CI)	p-value	Mean FC (95% CI)	p-value	Mean FC (95% CI)	p-value
Genistin	1.03 (−2.55 to 2.50)	0.999	1.09 (−3.14 to 2.96)	0.999	—	—	0.96 (−0.48 to 0.56)	0.999
Luteolin	0.96 (−2.48 to 2.56)	0.999	1.83 (−3.88 to 2.22)	0.997	—	—	0.71 (−0.23 to 0.81)	0.766
Polydatin	0.90 (−2.42 to 2.62)	0.999	1.88 (−3.93 to 2.16)	0.985	—	—	0.98 (−0.50 to 0.54)	0.999
Curcumin	1.13 (−2.65 to 2.39)	0.999	2.43 (−4.48 to 1.61)	0.822	—	—	0.70 (−0.22 to 0.83)	0.838
Apigenin	0.76 (−2.28 to 2.77)	0.912	2.05 (−4.10 to 2.00)	0.996	—	—	0.59 (−0.11 to 0.93)	0.337
Myricetin	0.74 (−2.26 to 2.78)	0.914	1.89 (−3.94 to 2.16)	0.999	—	—	0.77 (−0.29 to 0.75)	0.965
Isoliqu	0.95 (−2.47 to 2.57)	0.999	2.02 (−4.07 to 2.02)	0.991	—	—	0.49 (−0.03 to 1.01)	0.148
Ursolic acid	4.35 (−5.87 to −0.82)	*0*.*004*	0.64 (−3.04 to 3.77)	0.864	—	—	0.69 (−0.21 to 0.83)	0.532
EGCG	1.11 (−2.63 to 2.41)	0.999	1.81 (−3.86 to 2.24)	0.999	—	—	0.62 (−0.14 to 0.90)	0.442
Naringin	0.95 (−2.48 to 2.57)	0.999	1.09 (−3.14 to 2.95)	0.999	—	—	0.92 (−0.44 to 0.60)	0.999
Sulforaphane	0.92 (−2.44 to 2.60)	0.999	1.75 (−3.80 to 2.29)	0.993	—	—	0.25 (0.23 to 1.27)	*0*.*001*

Basal matrix metalloproteinase gene expression in human articular primary chondrocytes pre-treated with 10 µM of each bioactive for 6 hours. Cell isolates from three patients were used (*n* = 3) and each independent experiment performed in triplicate. Expression levels of *MMP1*, *MMP13*, *ADAMTS4* and *ADAMTS5* mRNA, normalised to *18S* were measured by RT-qPCR. Mean fold change (FC) to control, 95% confidence intervals (CI) are shown. One-way ANOVA, and all treatments were compared to control with Dunnett’s post-test corrected p-values shown.

IL1-induced *MMP1*, *MMP13*, *ADAMTS4* and *ADAMTS5* mRNA expression was consistently decreased by luteolin but did not reach statistical significance. Apigenin significantly decreased IL-1-induced *MMP13*, *ADAMTS4 and ADAMTS5* expression (mean fold change and 95% CI: 0.18 (0.09 to 1.55), p = 0.005, 0.11 (0.20 to 1.58), p = 0.0001 and 0.22 (−0.06 to 1.62), p = 0.018 respectively). Isoliquiritigenin significantly decreased the expression of IL-1-induced *MMP13*, *ADAMTS4* and *ADAMTS5* (mean fold change and 95% CI: 0.14 (0.14 to 1.59), p = 0.0005, 0.08 (0.15 to 1.69), p = 0.0001, and 0.11 (0.05 to 1.73), p = 0.001 respectively). Sulforaphane significantly decreased IL-1-induced expression of *MMP13* and *ADAMTS4* (mean fold change and 95% CI: 0.12 (0.15 to 1.61), p = 0.001, 0.03 (0.28 to 1.66), p = 0.0001), while showing a trend to decreasing both IL-1-induced *MMP1* and *ADAMTS5* (Table 1). Ursolic acid increased basal *MMP1* expression mean fold change 4.34 (95% CI: −5.87 to −0.82 p = 0.004) and sulforaphane inhibited basal *ADAMTS5* expression mean fold change 0.25 (95% CI: 0.23 to 1.27, p = 0.001) (Table 2). Three bioactives apigenin (API), isoliquiritigenin (ISO) and luteolin (LUT) were taken forwards, along with sulforaphane (SFN), as having activity regulating the expression of four key genes relevant to OA in HACs.

**Figure 1:**
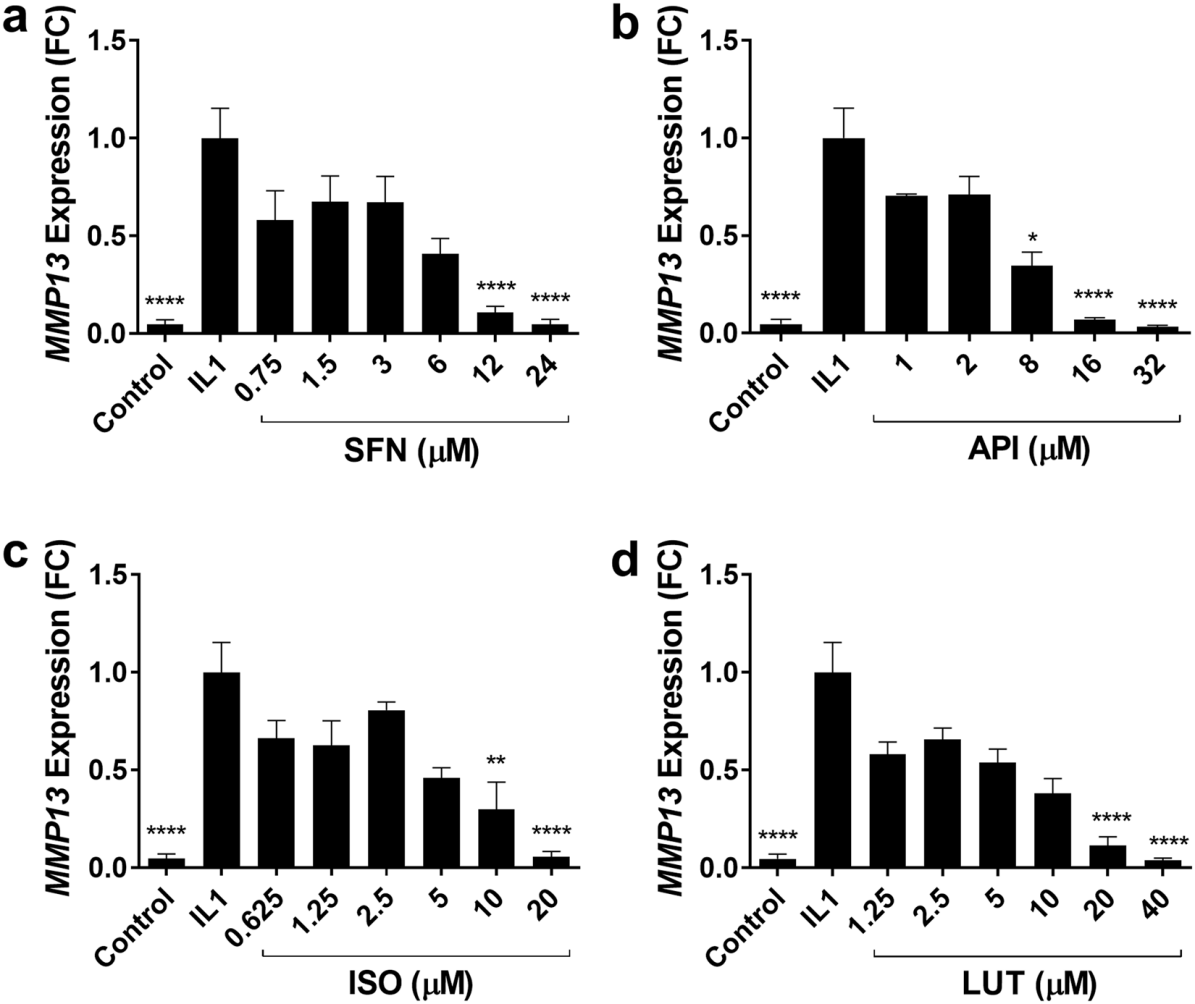
IL-1-induced *MMP13* gene expression in human primary articular chondrocytes. Four selected bioactives (**a**) sulforaphane (SFN), (**b**) apigenin (API), (**c**) isoliquiritigenin (ISO), (**d**) luteolin (LUT) were titrated against IL-1 treatment (5 ng/ml) in at least three independent isolates of human primary articular chondrocytes (*n* = 3) each performed in triplicate, for 6 h to establish a dose response. *MMP13* mRNA expression was measured using RT-qPCR and normalised to *18S*. Data is expressed as mean fold change (FC) ± SEM. One-way ANOVA, and all treatments were compared to IL-1 treatment with Dunnett’s post-test. Statistical significance is shown as *p < 0.05, **p < 0.01, ***p < 0.001, ****p = 0.0001.

We established a dose response for each bioactive against IL-1-induced *MMP13* expression in HACs using three separate patient cell isolates *n* = 3, each in triplicate conditions (Fig. 1). SFN reached significance at 12 µM and 24 µM mean fold change and 95% CI: 0.11 (0.53 to 1.26) p = 0.0001, and 0.05 (0.57 to 1.33) p = 0.0001 respectively (Fig. 1a). API reached significance at 8 µM mean fold change and 95% CI: 0.35 (0.36 to 0.95) p = 0.037, 16 µM 0.07 (0.65 to 1.22) p = 0.0001 and 32 µM 0.03 (0.68 to 1.25) p = 0.0001 (Fig. 1b). ISO was significant at 10 µM mean fold change and 95% CI: 0.30 (0.35 to 1.06) p = 0.002 and 20 µM 0.06 (0.59 to 1.30) p = 0.0001 (Fig. 1c). LUT was significant at 20 µM mean fold change and 95% CI: 0.11 (0.60 to 1.17) p = 0.0001 and 40 µM 0.04 (0.67 to 1.25) p = 0.0001 (Fig. 1d).

Hence, a wide screen of dietary-derived bioactives led us to focus on a subset of 10 compounds with further analyses highlighting 4 compounds, which dose-dependently inhibit IL-1-induced *MMP13* expression in primary human articular chondrocytes.

### Bioactives affect reactive oxygen species (ROS) in primary human chondrocytes

In order to investigate potential mechanism of action, direct antioxidant activity of the bioactives was measured in primary human articular chondrocytes (three separate patient cell isolates *n* = 3, each in triplicate). Primary chondrocytes were pre-treated for 30 minutes with the bioactives and then 100 µM tert-butyl hydrogen peroxide (tBHP) was added to induce cellular reactive oxygen species (ROS).

**Figure 2:**
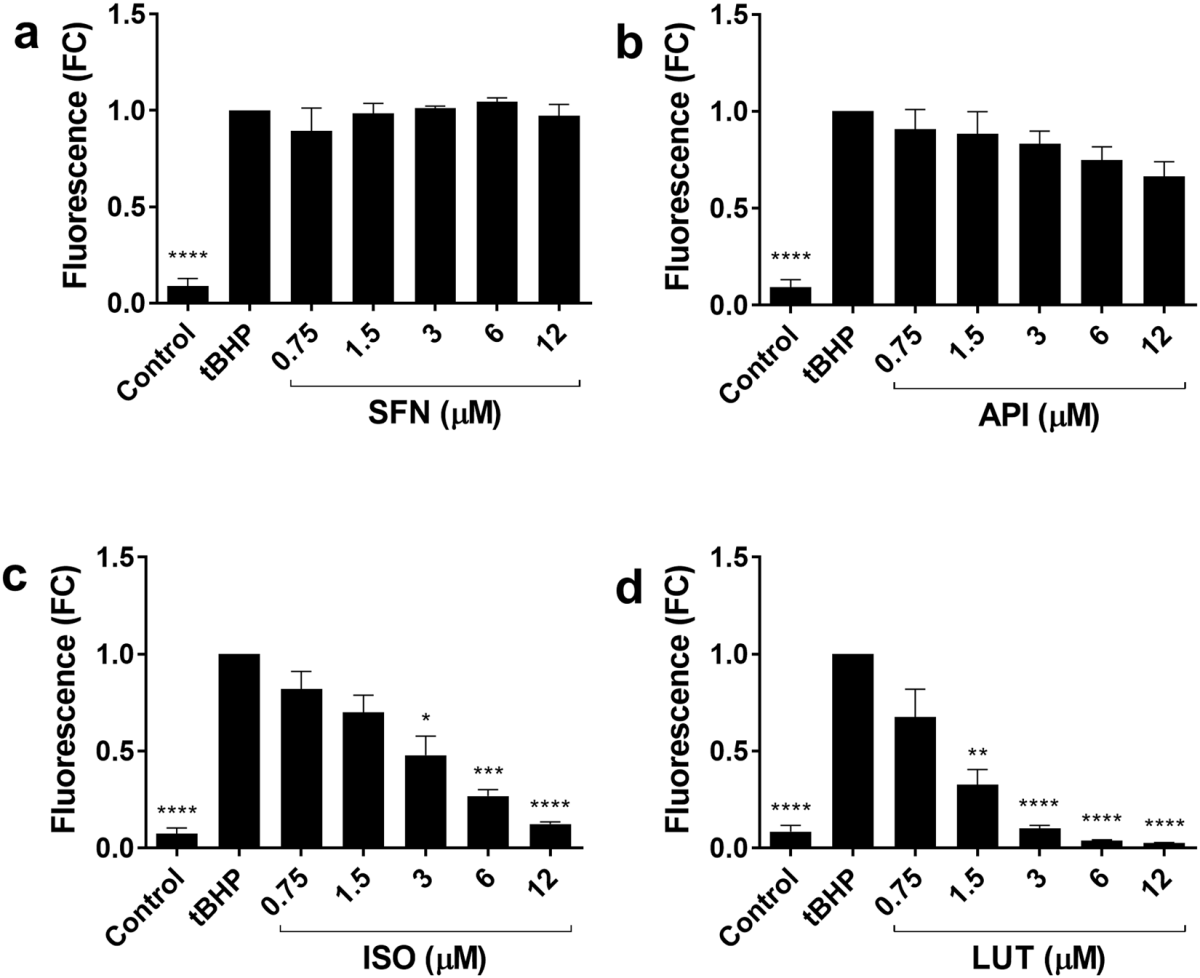
Reactive oxygen species (ROS) measured using the DCFDA assay in human primary articular chondrocytes. Cells were treated with 100 µM tert-butyl-hydroperoxide (tBHP) alone (4 h) ± pre-treatment with (**a**) SFN, (**b**) API, (**c**) ISO or (**d**) LUT at 0.75–12 µM for 30 mins. Data are from three independent isolates of human primary articular chondrocytes (*n* = 3), where each experiment was performed in triplicate. Data are shown as mean fold change (FC) to control ± SEM. One-way ANOVA, and all treatments were compared to IL-1 treatment with Dunnett’s post-test. Statistical significance is shown as: **p < 0.01, ***<0.001, ****p ≤ 0.0001.

ISO and LUT showed a dose dependent inhibition of tBHP-induced ROS that was statistically significant (Fig. 2). ISO inhibited ROS production: (3 µM mean fold change and 95% CI: 0.48 (0.26 to 0.78), p = 0.030, 6 µM 0.27 (0.47 to 0.99), p = 0.0005) and 12 µM 0.12 (0.62 to 1.14), p = 0.0001 (Fig. 2c). LUT inhibited ROS production: (1.5 µM mean fold change and 95% CI: 0.33 (0.41 to 0.94), p = 0.0035, 3 µM 0.10 (0.64 to 1.16), p = 0.0001, 6 µM 0.04 (0.70 to 1.22), p = 0.0001 and 12 µM 0.03 (0.71 to 1.24), p = 0.0001) (Fig. 2d). No effect was seen for SFN or API (Fig. 2a,b).

This showed that both ISO and LUT exert a direct antioxidant effect in chondrocytes, in contrast to SFN and API.

### Transcriptional signalling pathway analysis

**Figure 3:**
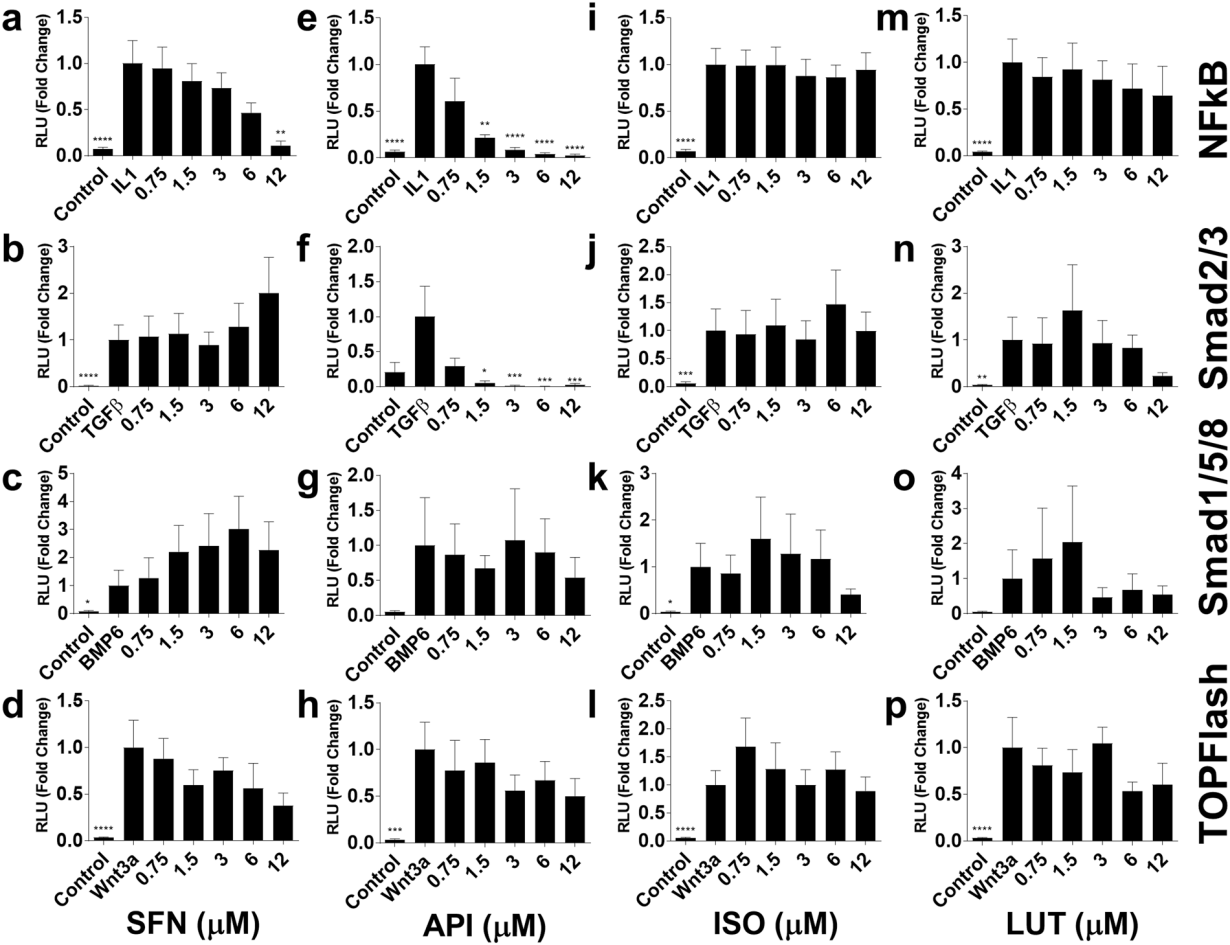
Transcriptional signalling pathway analysis. Luciferase reporter activity for IL-1/nuclear factor-kappa B, TGF-β/Smad2/3, BMP6/Smad1/5/8 and Wnt3A/TCF/Lef (TOPFlash), and using the SW1353 chondrosarcoma cell line in response to (**a**–**d**) SFN, (**e**–**h**) API, (**i**–**l**) ISO or (**m**–**p**) LUT treatment. Cells were treated with IL-1 (5 ng/ml, 2 h), TGF-β (1 ng/ml, 6 h), BMP6 (200 ng/ml, 24 h) and Wnt3a (100 ng/ml, 24 h). Values given are from at least three independent experiments (*n* = 3) where each experiment was performed in triplicate. Data are shown as mean fold change (FC) to control ± SEM. One-way ANOVA, and all treatments were compared to IL-1 treatment with Dunnett’s post-test. Statistical significance is shown as: *p < 0.05, **p < 0.01, ***p < 0.001, p ≤ 0.0001. RLU – relative light units (normalised to control plasmid).

A number of signalling pathways play a role in cartilage homeostasis and OA. Luciferase reporters for IL-1/nuclear factor-kappa B, TGF-β/Smad2/3, Wnt3A/TCF/Lef, and BMP6/Smad1/5/8 signalling pathways were used to measure transcriptional activity in response to SFN, API, ISO and LUT treatment (Fig. 3). Data are from a minimum of three independent experiments (*n* = 3) each performed in triplicate.

SFN dose dependently inhibited IL-1-induced NFκB signalling reaching significance at 12 µM mean fold change and 95% CI: 0.11 (0.8 to 1.7), p = 0.003 (Fig. 3a) and Wnt3A/TCF/Lef signalling (not significant) (Fig. 3d). SFN showed trends of increasing TGF-β-induced Smad2/3 and BMP6-induced Smad1/5/8 signalling (not significant) (Fig. 3b,c).

API strongly inhibited IL-1/NFκB (1.5–12 µM mean fold change and 95% CI: 0.22 (0.30 to 1.26), p = 0.009, 3 µM 0.09 (0.43 to 1.40), p = 0.0001, 6 µM 0.04 (0.48 to 1.44), p = 0.0001, 12 µM 0.03 (0.45 to 1.49), p = 0.0001) (Fig. 3e) and TGF-β/Smad2/3 signalling at 1.5–12 µM (1.5 µM mean fold change and 95% CI: 0.06 (0.22 to 1.67), p = 0.002, 3 µM 0.02 (0.26 to 1.70), p = 0.0008, 6 µM 0.01 (0.27 to 1.71) p = 0.0001, 12 µM 0.03 (0.19 to 1.76), p = 0.001) (Fig. 3f). In contrast API had no effect on BMP6/Smad1/5/8 (Fig. 3g) and decreased Wnt3A/TCF/Lef activity weakly in a dose dependent manner (not significant) (Fig. 3h).

We observed a small decrease in BMP6-induced Smad1/5/8 signalling at 12 µM (not significant) with ISO treatment (Fig. 3k) but no effect on the IL-1/NFκB, TGF-β/Smad2/3 or Wnt3A/TCF/Lef reporters (Fig. 3i,j,l).

LUT showed a weak dose dependent inhibition of IL-1/NFκB signalling (not significant) (Fig. 3m) and small decreases at the high concentrations for TGF-β/Smad2/3 (Fig. 3n), BMP6/Smad1/5/8 (Fig. 3o) and Wnt3A/TCF/Lef (Fig. 3p) reporters (not significant).

Thus, SFN and API both inhibit NFκB signalling, API also inhibits Smad2/3 signalling. SFN also shows weak induction of Smad2/3 and Smad1/5/8 signalling and repression of canonical Wnt signalling. There was minimal impact of the four compounds on other signalling pathways tested.

### Bioactives reduce cartilage tissue degradation *in vitro*

**Figure 4:**
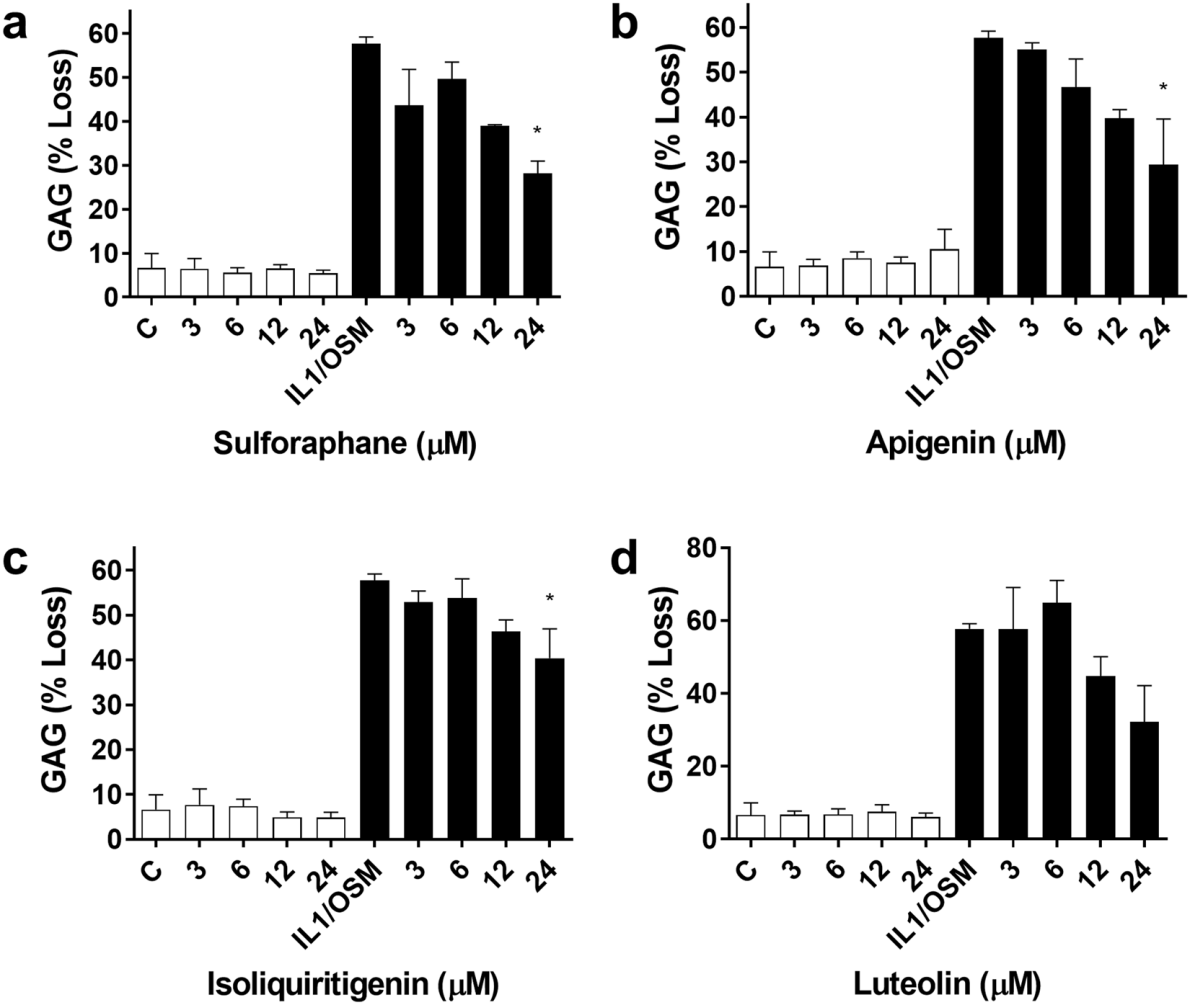
Cartilage tissue degradation *in vitro*. Aggrecan loss measured using 1,9-dimethyl-methylene blue assay from bovine cartilage explants in response to (**a**) SFN, (**b**) API, (**c**) ISO or (**d**) LUT treatment (open bars), +IL-1/OSM at 0.5 ng/ml and 5 ng/ml respectively (black bars). Values given are from three independent experiments (*n* = 3) and each performed in triplicate. Data are shown as mean percent loss of total glycosaminoglycan (GAG) ± SEM. One-way ANOVA, and all treatments with cytokines were compared to IL-1/OSM treatment alone with Dunnett’s post-test. Statistical significance is shown as: *p < 0.05.

In order to examine the impact of bioactivity on cartilage homeostasis, bovine nasal cartilage explants were induced to degrade using inflammatory cytokines IL-1 and OSM in three independent experiments (*n* = 3) each performed in triplicate. Cartilage was treated with 0–24 µM SFN, API, ISO or LUT ±IL-1 and OSM and the degradation product of aggrecan (glycosaminoglycan) was measured using 1,9-dimethyl-methylene blue assay (Fig. 4). All four bioactives showed dose-dependent patterns inhibiting cartilage degradation: IL1/OSM treatment mean % GAG loss and 95% CI: 57.74 (41.1 to 61.11). SFN (24 µM mean % loss and 95% CI: 28.13 (12.06 to 47.16), p = 0.0019) and significant linear trend (slope = −0.07, p = 0.0009, r^2^ = 0.78) (Fig. 4a). API (24 µM mean % loss and 95% CI: 29.45 (5.99 to 50.6), p = 0.022) with significant linear trend (slope = −0.08, p = 0.0043, r^2^ = 0.90) (Fig. 4b). ISO (24 µM mean % loss and 95% CI: 40.37 (1.40 to 33.34), p = 0.049) and significant linear trend (slope = −0.04, p = 0.013, r^2^ = 0.88) (Fig. 4c). Similarly, LUT (24 µM mean % loss and 95% CI: 32.25 (−5.96 to 56.94), p = 0.080), and showed a significant linear trend (slope = −0.07, p = 0.022, r^2^ = 0.64) (Fig. 4d).

All compounds show a statistically significant dose-response (i.e. significant decrease across the dose range even though individual lower concentrations are not significant on their own). SFN, API and ISO show statistical significance in preventing IL1/OSM-induced GAG release from cartilage explants at the highest dose tested.

### Kinase signalling pathway analysis

It is likely that compounds alter intracellular kinase signalling, therefore we used kinase arrays to identify potential regulation of this in response to IL-1 stimulation (5 ng/ml, 10 mins) by SFN or ISO (10 µM) in human primary chondrocytes. SFN inhibited IL-1 phosphorylation of Akt1/2/3 (s473), c-jun, CREB, ERK1/2, PRAS40, WNK1 (all >25% decrease). ISO inhibited Akt1/2/3 (s473), β-catenin, EGF-R (>25% decrease). SFN reduced basal levels of phosphorylation (>50%) compared to control of c-jun, CREB, ERK1/2, HSP27, JNK1/2/3, p27 and p38α *n* = 1 (Supplementary data S2).

### Bioactive synergism for inhibition of *MMP13* expression in primary chondrocytes

Since foods are rarely eaten alone, the synergism between compounds with respect to IL1-induced *MMP13* expression was determined. All synergy experiments used cell isolates from at least three separate patients *n* = 3, each performed in duplicate. The dose-effect relationship parameters of each single bioactive SFN, API, ISO and LUT against IL1-induced *MMP13* mRNA expression in HACs were determined. The rank order of potency based on the IC_50_ values were: ISO > SFN > LUT > API in HACs (Table 3).

**Table 3:** Dose-effect relationship parameters of SFN, API, ISO and LUT individually against IL1-induced *MMP13* mRNA expression in primary chondrocytes.

Bioactive	D_*m*_	*m*	*r*
SFN	3.33	1.43	0.75
API	6.76	2.54	0.93
ISO	2.89	1.24	0.73
LUT	3.80	1.32	0.84

IC_50_ concentration (D_*m*_) is shown in µM. The slope (*m*) and linear correlation coefficient (*r*) of the median-effect plot are given. Values are the mean of three human articular primary chondrocyte isolates (*n* = *3*) and each experiment contained six concentrations for each bioactive, in duplicate. Gene expression was measured using RT-qPCR and normalised to *18S*.

The combination index (CI) values of bioactive combinations against IL1-induced *MMP13* mRNA expression in HACs was measured. Combination ratios were based on IC_50_ values rather than absolute concentrations. Each component had the equipotent combination ratio of: SFN:API:ISO:LUT (S:A:I:L) = 0.75:1:0.625:1.25 µM. Synergism was only seen in the SFN/ISO (SI) combination (mean CI: 0.724). Nearly additive combinations were SA (mean CI: 1.02) and AI (mean CI: 1.06) where SA ≥ AI. All three (and four) bioactive combinations were antagonistic. Rank order for antagonism was SAIL ≥ IL ≥ LSA ≥ ILS ≥ AIL ≥ SL > SAI > AL (Table 4). Note assigned symbols for the combination indices are taken from Chou^32^.

**Table 4:** Bioactive combination indices of SFN, API, ISO and LUT for *MMP13* expression in primary chondrocytes.

Bioactive Combo	CI Values					Assigned symbol
	ED_50_	ED_75_	ED_90_	ED_95_	mean CI	
SA	0.706	0.879	1.127	1.356	1.017	±
SI	0.872	0.758	0.661	0.604	0.724	++
SL	1.066	1.404	1.851	2.235	1.639	—
AI	1.213	1.072	0.997	0.975	1.064	±
AL	0.887	1.008	1.189	1.361	1.111	-
IL	3.111	2.208	1.569	1.244	2.033	—
SAI	1.066	1.161	1.311	1.454	1.248	–
AIL	1.919	1.754	1.663	1.642	1.744	—
ILS	1.550	1.712	1.896	2.034	1.798	—
LSA	1.753	1.836	1.978	2.119	1.922	—
SAIL	1.368	1.770	2.357	2.917	2.103	—

Combination Index (CI) values of bioactive combinations against IL1-induced *MMP13* mRNA expression in human articular primary chondrocytes (*n* = 3) measured by RT-qPCR and normalised to *18S*. Combination ratios were based on IC_50_ values. Each bioactive component had a combination ratio of: S:A:I:L = 0.75:1:0.625:1.25 µM. Assigned symbols are: nearly additive (±), moderate synergism (++), slight antagonism (-), moderate antagonism (–), antagonism (—)^32^. CI = Combination index.

The dose reduction index (DRI) values and relationship to CI values in synergistic combinations were determined (Table 5). The two-bioactive combination SFN + ISO show a robust dose reduction from ED_50_ to ED_95_. This favourable dose reduction was also seen for SFN + API at ED_50_ to ED_75_ and API + ISO at ED_90_ to ED_95_ even though the average unweighted CI values suggest a nearly additive effect (Table 4).

**Table 5:** Dose reduction Index (DRI) values for *MMP13* expression in relation to combination index (CI) values for synergistic combinations of SFN/API, SFN/ISO and API/ISO in primary chondrocytes.

Combination	DRI Values at:				CI Values at:			
	ED_50_	ED_75_	ED_90_	ED_95_	ED_50_	ED_75_	ED_90_	ED_95_
S+	1.418	3.292	7.641	13.549	0.706	0.879	1.127	1.356
A (0.75:1)	1.891	4.389	10.188	18.066				
S+	1.479	2.930	5.808	9.248	0.872	0.758	0.661	0.604
I (0.75:0.625)	1.232	2.442	4.840	7.706				
A+	3.328	5.781	10.043	14.622	1.213	1.072	0.997	0.975
I (1:0.625)	2.080	3.613	6.277	9.139				

The DRI describes the fold reduction in bioactive concentration needed in a synergistic combination at each effect level shown (compared with each bioactive alone). Gene expression was measured by RT-qPCR, normalised to *18S*, from three independent experiments, *n* = 3. Synergism (CI < 1), additivity (CI = 1), antagonism (CI > 1).

A polygonogram summary of two-bioactive combination interactions, for the four bioactives is shown (Supplementary data S3). The combination of SFN and ISO show synergistic activity for reducing IL-1 induced *MMP13* expression in primary chondrocytes at each effect level. The average combination indices for SFN/API and API/ISO were ‘nearly additive’ however, it is noted that the SFN/API combination shows slight to moderate synergism at lower effect levels.

### SFN/ISO combination regulates the expression of other key genes in OA

**Figure 5:**
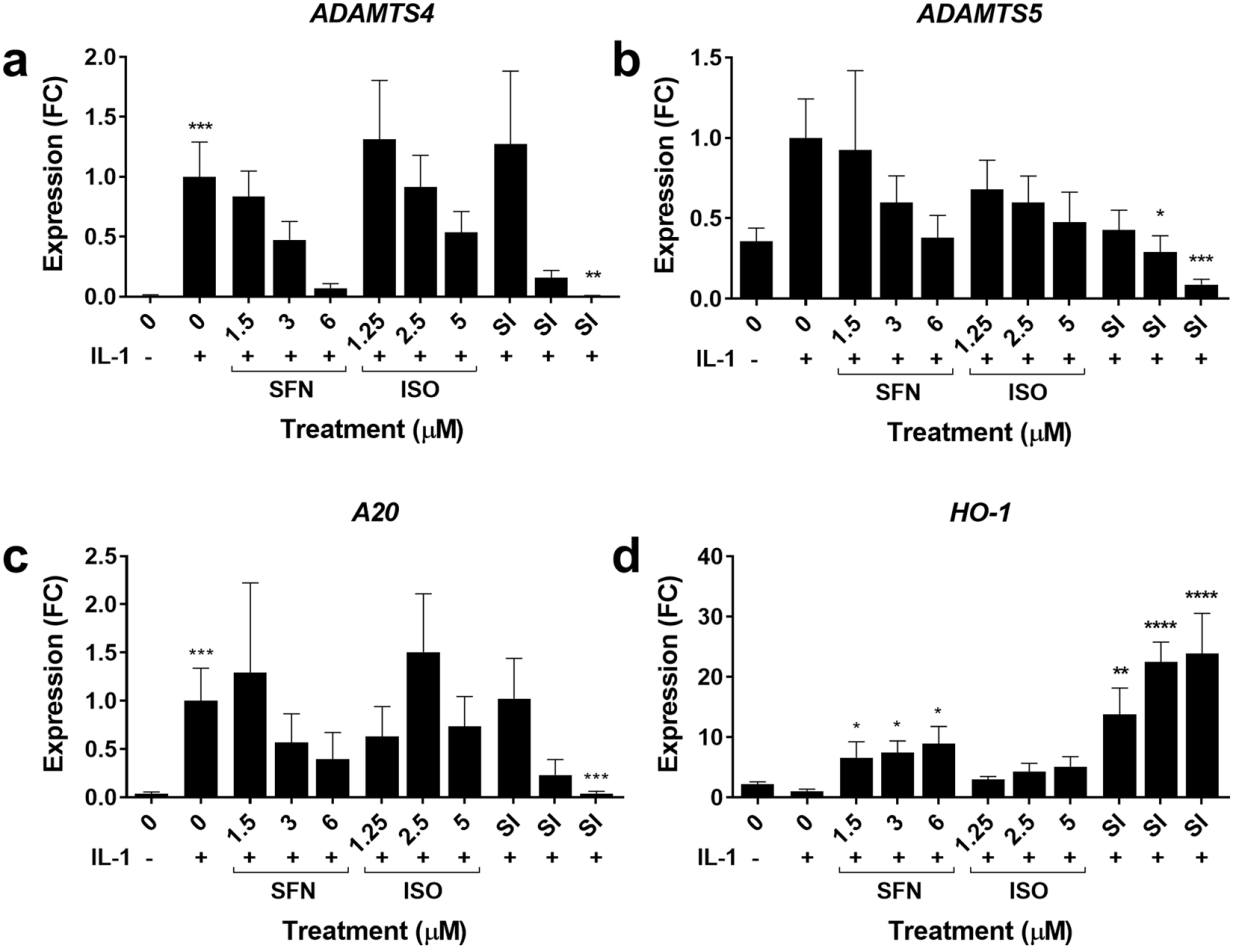
Cytokine-induced expression levels of key genes in osteoarthritis with SFN/ISO combination treatment. IL1-induced gene expression of *ADAMTS4*, *ADAMTS5*, *HO*-*1* and *A20* in human articular primary chondrocytes (HACs). HACs were pre-treated with SFN or ISO individually and in the equipotent SFN:ISO combination ratio: 0.75:0.625 µM for 30 mins followed by IL1 (5 ng/ml) 6 hours. Gene expression was measured in at least three independent isolates of human primary articular chondrocytes (*n* = 3) by RT-qPCR and normalised to *18S*. Data is expressed as mean fold change (FC) from IL-1 ± SEM. One-way ANOVA, and all treatments were compared to IL-1 treatment with Dunnett’s post-test. Statistical significance is shown as: *p < 0.05, **p < 0.01, ***p < 0.001, ****p ≤ 0.0001.

In order to investigate the potential of the SFN/ISO combination to interact synergistically to regulate other relevant genes, we used RT-qPCR to measure the expression of proteases *ADAMTS4*, *ADAMTS5*, the Nrf2-induced indirect antioxidant *HO*-*1*, and the early NFκB response gene and regulator *A20*. We used the synergism sample set at equipotent IC_50_ ratios of 0.75:0.625 µM where *n* = 3 (Fig. 5).

IL-1-induced *ADAMTS4* was inhibited by SFN and ISO singly (not significant) and in combination (SI (1.5:1.25 µM) mean fold change 1.27 (95% CI: −1.39 to 0.84), p = 0.99), (SI (3:2.5 µM) mean fold change 0.16 (95% CI: −0.27 to 1.96), p = 0.014), (SI (6:5 µM) mean fold change 0.005 (95% CI −0.12 to 2.11), p = 0.034) (Fig. 5a).

IL-1-induced *ADAMTS5* was inhibited by SFN and ISO singly (not significant) and in combination (SI (1.5:1.25 µM) mean fold change 0.57 (95% CI: −0.26 to 1.40), p = 0.73), (SI (3:2.5 µM) mean fold change 0.71 (95% CI: −0.12 to 1.54), p = 0.007), (SI (6:5 µM) mean fold change 0.09 (95% CI: 0.08 to 1.74), p = 0.0001) (Fig. 5b).

IL-1-induced *A20* was inhibited by SFN singly (not significant) and in combination with ISO (SI (1.5:2.5 µM) mean fold change 1.02 (95% CI: −1.68 to 1.63), p = 0.997), (SI (3:2.5 µM) mean fold change 0.23 (95% CI: −0.89 to 2.42), p = 0.16, (SI (6:5 µM) mean fold change 0.04 (95% CI: −0.69 to 2.62), p = 0.004) (Fig. 5c).

*HO*-*1* was induced by SFN singly and in combination with ISO compared to control (SFN 1.5 µM mean fold change 6.59 (95% CI: −16.31 to 7.58), p = 0.025), (3 µM mean fold change 7.44 (95% CI: −17.17 to 6.72), p = 0.022), (6 µM mean fold change 8.96 (95% CI: −18.69 to 5.20), p = 0.012). ISO alone did not induce *HO-1* expression. In combination, (SI (1.5:1.25 µM) mean fold change 13.74 (95% CI −23.47 to 0.42), p = 0.0018), (SI (3:2.5 µM) mean fold change 22.5 (95% CI −32.22 to −8.33), p = 0.0001), (SI (6:5 µM) mean fold change 23.86 (95% CI: −33.59 to −9.70), p = 0.0001) (Fig. 5d).

Whilst mathematical synergism for these genes wasn’t determined as for *MMP13* above, the combination of SFN and ISO together was clearly more effective that either compound alone, confirming the utility of the approach.

## Discussion

A strategy to prevent or treat OA using dietary modification, supplementation or the development of functional foods is clearly attractive. It also has wide public health implications particularly in a chronic disease, which is increasing in prevalence with no disease-modifying treatments currently available. There is a need to understand the mechanism by which diet-derived bioactives can impact upon the joint, both *in vitro* and *in vivo*. This is vital for the design of appropriate intervention trials and therefore the translation to treatment in man.

Our strategy was to screen diet-derived compounds for their ability to inhibit either basal or IL1-induced *MMP13* expression since this identified SFN, which was then shown to be chondroprotective in animal models of OA^16^. Further we hypothesised that if bioactives regulate gene expression through differing mechanisms, then they are more likely to be synergistic. Of the 96 bioactives in our initial screen for expression of key OA metalloproteinase genes, the four most efficacious compounds were SFN, API, ISO and LUT.

The effect of SFN on metalloproteinase expression and cartilage degradation was in line with earlier work^16,33,34^ and unsurprisingly, had no direct effect on ROS production since it is well documented by others that SFN is an indirect antioxidant^35^. SFN affected each of the four cytokine-stimulated pathways; IL-1, TGFβ, BMP6 and Wnt3A with differing efficacy. Of the four pathways analysed, SFN interacted most strongly with the IL-1/NFκB pathway, likely in part as a function of SFN’s known indirect antioxidant and anti-inflammatory activities, which we have reported previously^16^. SFN also inhibited Wnt3a canonical signalling, an observation seen previously in breast cancer stem cells^36^ but diverged to enhance both TGFβ/Smad signalling, which is reported similarly in colorectal cancer cells though not in a murine model of muscle fibrosis^37,38^), and BMP6 signalling. Moreover, BMP6/Smad1/5/8 pathway is reported to induce HO-1 expression^39,40^ and so SFN could be viewed overall as a dietary modulator of cartilage homeostasis. This is reinforced by its efficacy in animal models of OA^16,17,19^. Since SFN is a well-known activator of the Nrf2 signalling pathway^41^ and though not measured here, this may be a significant contributing mechanism of action.

Our data support previous studies where API inhibited IL-1-induced metalloproteinase expression^25,26^, and is reflected by the decrease in glycosaminoglycan loss also seen by Lim *et al*.^26^ in rabbit articular cartilage. API showed remarkably strong and targeted activity for inhibiting IL-1/NFκB and TGF-β/Smad2/3 signalling pathways while having no effect on BMP6/Smad1/5/8 signalling or only a weak inhibitory effect on the Wnt3A/TCF/Lef pathway. Inhibition of NFκB signalling by API agrees with some earlier studies^42,43^ (though not Lim *et al*.^26^), as does the inhibition of TGFβ/Smad signalling in prostate cancer cell lines^44^. Conversely, Zhang *et al*. demonstrate apigenin activation of the TGFβ/Smad pathway in skin fibroblasts to induce collagen synthesis, albeit at a later time point (12 hours)^45^. Similarly for SFN, we did not observe direct antioxidant activity for API in primary chondrocytes though the literature describes varied outcomes and mechanisms for API including antioxidant activity that may be cell type dependent. Yokomizo & Moriwaki describe a lack of radical scavenging activity for API in Caco-2 cells^46^, and API is described as a driver of ROS production^47^, which we did not observe in chondrocytes (data not shown). Conversely, Crasci *et al*. describe free radical scavenging activity for both API and LUT in chondrocytes^48^.

Our data on both metalloproteinase expression and explant cartilage degradation support a recent study demonstrating *in vivo*, the potential for ISO (administered by intraperitoneal injection) to protect articular cartilage, where the authors also show protection of subchondral bone tissue in the joints of mice^28^. This followed another recent report of ISO inhibition of IL-1-induced metalloproteinase expression in rats^29^. Interestingly and in contrast to SFN and API, ISO showed a consistent dose dependent inhibition of tBHP-induced ROS production indicating some direct anti-oxidant activity. We did not find evidence to support an interaction of ISO with any of the reporters tested and suggest an interaction with Nrf2 to be more likely^30,49,50^.

LUT showed a similar activity profile to ISO overall despite being structurally more similar to API (one hydroxyl group difference in the B ring). ISO inhibition of tBHP-induced ROS production was striking in relation to other activity measures and supports an earlier report in Caco-2 cells^46^, where no effect by API was also seen. There are a number of reports of LUT that describe reactive oxygen and nitrogen species scavenging, transition metal chelation and antioxidant enzyme induction and is reviewed in detail elsewhere^21^. Nonetheless, we observed no marked interaction with the pathway reporters excepting the weak interaction with the IL-1/NFκB reporter. Despite the repression of metalloproteinase expression by LUT (also seen by others^22,51^) that was borne out in our cartilage degradation assay, it was surprising to observe an antagonistic relationship for reducing *MMP13* expression between LUT and each of the remaining bioactives.

It is possible that the anti-inflammatory/cytoprotective activities of SFN and antioxidant activities of ISO culminate in the synergistic interaction to reverse IL-1-induced *MMP13* expression that we have identified. The nearly additive relationship of SFN and API could be explained by their similar activity profiles, which may saturate signalling pathways, whilst neither having direct antioxidant activities in our hands. However, this may be simplistic since the interaction profile of SFN/API with *MMP13* expression differs substantially at different effect levels, being mild to moderately synergistic at lower effect levels. A high effect level, though clinically desirable, is not a necessity in terms of OA prevention or treatment.

Our data suggest antagonistic interactions (i.e. the effect of both compounds is less than the additive effect of the individual compounds) between SFN/LUT and ISO/LUT, which are more difficult to explain. Interestingly all combinations with 3 or more bioactives were antagonistic, except for the SFN/API/ISO combination, which was only moderately antagonistic, possibly negated by the SFN/ISO interaction. More broadly speaking, food matrices or food-drug interactions present a far more complicated milieu.

We also assayed the Nrf2-induced HO-1 gene and the NFκB-induced A20 gene, both readouts of key pathways in OA, where treatment with both SFN and ISO showed a greater effect than the additive effects of the single compounds.

We have clearly identified dietary-bioactives with the strong potential to be chondroprotective *in vivo* and with SFN and ISO displaying synergy. The concentrations of bioactives in our *in vitro* assays are either equivalent or higher than reported plasma concentrations after consumption (e.g.^52–54^. However, compounds or their metabolites can accumulate with repeated consumption, or be concentrated intracellularly^55^. The potential for functional foods or supplementation also increases the achievable concentration *in vivo*. It is worth noting that dietary compounds undergo extensive metabolism following ingestion and even when metabolism is well established, the mix of parent compound and metabolites that act upon the articular joint is unknown. It is also likely that the metabolites produced upon ingestion may be more active than the parent compound^56^. The only way to understand this is to perform feeding experiments in animal models of disease using chemically-defined foods as we have done for SFN alone^16^. *In vivo* experiments also enable a comparison of mechanism-of-action with the *in vitro* and *ex vivo* analyses. This is the direction of our future research in this area.

We conclude that diet-derived bioactives may be important modulators of cartilage homeostasis and synergistic relationships between bioactives (potentially SFN/ISO) in primary chondrocytes may have a role in chondroprotection and counterbalancing inflammation.

## Materials and Methods

### Materials

SFN was from Toronto Research chemicals, CAN. All other bioactives were obtained from Stratech, UK. All cytokines were obtained from Firstlink Ltd (UK) except Wingless-Type MMTV Integration Site Family 3A (WNT3A), which was obtained from R&D Systems. The Proteome Profiler Array (cat: ARY003B) was obtained from R&D Systems, UK.

### Methods

A flow diagram to summarise the experimental process is shown in Supplementary Information.

### Cell culture and treatments

The human chondrosarcoma SW1353 cell line was purchased from ATCC and the C28/I2 cell line was a kind gift from Mary Goldring, HSS Research Institute (USA). Primary chondrocytes were obtained as described previously^16^. All experimental protocols were approved by NRES Committee East of England (ref: 08/h0304/85+5). All tissue donors gave informed written consent. All methods were performed in accordance with relevant laboratory guidelines and institutional regulations. All treatments included 30 min pre-incubation with bioactive compound followed by cytokine treatment. Cells were propagated in in Dulbecco’s modified Eagles medium (DMEM, GlutaMAX) with 10% v/v foetal calf serum and 1000IU/penicillin, 100 µg/ml streptomycin, at 37 °C, 5% CO_2_.

### Complementary DNA (cDNA) synthesis and reverse transcription–quantitative polymerase chain reaction (RT-qPCR)

Whole cell lysates were harvested with Cells-to-cDNA II Cell Lysis Buffer (Ambion). Lysates were treated with DNase I (Ambion) and reverse transcribed in a total volume of 20 µl, using 200 ng random primers and 100 units Moloney murine leukaemia virus reverse transcriptase (Invitrogen), according to the manufacturer’s instructions, in the presence of 40 units RNasin (Promega). Relative quantification of genes was performed using an ABI Prism 7500 Sequence Detection System (Applied Biosystems). PCRs used 5 µl of reverse-transcribed RNA (a 10-fold dilution of cDNA was used for 18S analyses). The MMP and ADAMTS primers and probes were previously described^57,58^. The primers and probes for *HO*-*1*, *A20* were designed using the Universal Probe Library (Roche). Gene expression was normalised to ribosomal *18S* and relative quantification was calculated using 2^−ΔCt^.

### Gene promoter reporter assays

SW1353 cells were plated at 6 × 10^3^ cells/well in 96-well plates and left to adhere overnight. At least three independent experiments were carried out each performed in triplicate (*n* = 3). Transfections were carried out using 150 ng reporter plasmid DNA and 50 ng renilla control plasmid and Lipofectamine 2000 (Fisher Scientific) for 24 hours. Cells were synchronised by serum starvation overnight, prior to treatments. Optimal time and cytokine concentrations were determined for each reporter individually and used in all downstream experiments. Cytokine treatments were: interleukin-1 (IL1) (5 ng/ml) 2 hours; transforming growth factor beta (TGFβ) (1 ng/ml), 6 hours; bone morphogenic protein 6 (BMP6) (200 ng/ml), 24 hours; wingless-type MMTV integration site family 3A (WNT3a) (100 ng/ml), 24 hours. Luminescence was measured using the Dual-Glo® Luciferase Assay System (Promega) and EnVision Multilabel Plate Reader (PerkinElmer). pGL3 BRE Luciferase was a gift from Martine Roussel & Peter ten Dijke (Addgene plasmid #45126)^59^, pGL4.32[*luc2P*/NFκB-RE/Hygro] (Promega), pGL3[CAGA-*luc*] was a kind gift from Jean-Michel Gauthier^60^, M50 Super 8x TOPFlash was a gift from Randall Moon (Addgene plasmid #12456)^61^.

### Reactive Oxygen Species (ROS) Analysis

ROS were detected using 2′,7′-dichlorofluorescin diacetate (DCFDA Cellular ROS Detection Assay Kit from Abcam (cat: ab113851)) according to the manufacturer’s instructions. Briefly, primary chondrocytes were plated at 8 × 10^3^ cells per well in 96-well plates. The experiment was repeated three times using three different patient cell isolates (*n* = 3), each performed in triplicate. Treatments were 1.5–12 µM SFN, API, ISO or LUT pre-treatment for 30 mins ± (100 µM) tert-Butyl hydroperoxide (tBHP) for 4 hours. ROS levels were measured at Ex/Em 492/530 nm using EnVision Multilabel Plate Reader (PerkinElmer).

### *In vitro* cartilage degradation assays

Cartilage explants were pre-treated with 0–24 µM SFN, API, ISO or LUT. Cytokines IL-1 and OSM (0.5 ng/ml and 5 ng/ml respectively) were added to induce cartilage breakdown. All treatments were performed in triplicate and the experiment performed three times (*n* = *3*). Remaining cartilage was papain-digested overnight at 65 °C. Glycosaminoglycan (GAG) was measured in the media and digested cartilage as described previously^62,63^.

### Kinase signalling pathway analysis

Primary chondrocytes were plated at 3 × 10^6^ cells per well in 150 mm plates and allowed to adhere overnight. Chondrocytes were treated with IL-1β (5 ng/ml), for 10 mins with or without SFN, API or ISO (10 µM) pre-treatment (30 mins). Cell lysates were prepared and applied to Proteome Profiler Array (R&D Systems) according to the manufacturer’s instructions. Phosphorylation levels were semi-quantified using ImageJ software.

### Cytotoxicity in HACs

HACs were treated with 10 µM of each bioactive for 24 hours in three independent cell isolates (*n* = 3). Lactate dehydrogenase (LDH) was measured in the culture media using the CytoTox 96® NonRadioactive Cytotoxicity Assay (Promega) according to the manufacturer’s instructions. Total LDH was measured by lysing cells. Absorbance was measured at 492 nm using an EnVision Multilabel Plate Reader (PerkinElmer) (Supplementary data S4).

### Statistical testing

Fold change values were log transformed and one-way analysis of variance (ANOVA) with Dunnett’s post-test, were performed using GraphPad Prism version 7.03 for Windows to obtain adjusted p-values. Mean fold change and confidence intervals are reported for fold change values. p < 0.05 was considered significant. Synergism was analysed using the Chou & Talalay method^64^ and CompuSyn v1.0 software. http://www.combosyn.com/index.html.

## Electronic supplementary material

Supplementary Information

## Data Availability

The datasets generated during and/or analysed during the current study are available from the corresponding author on reasonable request.
