# Supplementary material for: Identifying chondroprotective diet-derived bioactives and investigating their synergism
Source: Sci Rep. 2018 Nov 21;8:17173. doi: 10.1038/s41598-018-35455-8 (PMC6249298; doi:10.1038/s41598-018-35455-8)
Supplement: Supplementary file 1 — Supplementary Information [file 41598_2018_35455_MOESM1_ESM.pdf]

Title: Identifying chondroprotective diet-derived bioactives and investigating their synergism

[1]Rose K Davidson Ph.D & [1]Jonathan Green Ph.D, [1]Sarah Gardner BSc, [2]Yongping Bao Ph.D, [2]Aedin Cassidy Ph.D, [1]Ian M Clark* Ph.D.

Biomedical Research Centre: [1]School of Biological Sciences, [2]Norwich Medical School, Department of Nutrition, Norwich Research Park, University of East Anglia, Norfolk, UK, NR4 7TJ.

This work was funded by the BBSRC Diet and Health Research Industry Club grants BB/J500112/1, BB/L025485/1.

Supplementary Information:

- **List of bioactives screened**
- **Experimental process**

Supplementary Data:

- **S1 - Heat Map**
- **S2 – Kinase signalling**
- **S3 – Two-bioactive polygonogram**
- **S4 – Cytotoxicity**

Supplementary Information

	Compounds	Common sources
1	Capsaicin	Chilli peppers
2	Sclareol	Salvia sclarea
3	(-)-Hupersine A	Huperzia serreta
4	(+)-Usniacin (D-Usnic acid)	Lichen
5	3-Indolebutyric acid (IBA)	Maize
6	4-Methylumbeliferone (4-MU)	Rice
7	Aesculin	Conkers (horse chestnut)
8	Aloe-emodin	Aloe vera
9	Sclareolide	Salvia sclarea
10	shikimic acid	American sweetgum
11	Apigenin	Parsley, celery, coffee
12	Silibinin	Milk thistle
13	Arbutin	Bear berries
14	Artemether	Sweet worm wood
15	Artesunate	Sweet wormwood
16	Asiatic acid	Gotu kola
17	Silymarin	Milk thistle
18	Baicalein	Blue and common skullcap
19	Sinomenine	Sinomenium Actum
20	Bergenin	Bergenia ciliata
21	Berberine Hydrochloride	Berberis
22	Sitosterol	Pecan
23	Bilobalide	Ginkgo biloba
24	Stigmasterol	Soy
25	Caffeic acid	Cucumber
26	Chlorogenic acid	Coffee
27	Chrysin	Honeycomb and passion flower
28	Tangeretin	Tangerine
29	Theobromine	Cocoa bean
30	Troxerutin	Styphnolobium japonicum
31	Ursolic acid	Apples
32	Cytisine	Gymnocladus dioica
33	Daidzin	Soy
34	Dihydroartemisinin	Sweet wormwood (tea)
35	Diosgenin	White yam
36	Vanillylacetone	Ginger
37	Diosmin	Peppermint
38	DL-Carnitine hydrochloride	Beef
39	Ecdysone	Cordyceps, sinensis
40	Emodin	Rhubarb
41	Enoxolone	Liquorice
42	Ergosterol	Maize
43	Fisetin	Strawberries
44	Formononetin	Red clover
45	Fumalic acid	Asafoetida
46	Genistin	Soy
47	Glycyrrhizic acid	Liquorice
48	Hydroxytryptophan	Turkey
49	Aloin (Barbaloin)	Aloe vera
50	Gramine	Arundo donax
51	Ammonium Glycyrrhizinate	Liquorice
52	Gynostemma Extract	Gynostemma pentaphyllum

	Compounds	Common sources
53	Hesperetin	Black peppermint
54	Hesperedin	Orange
55	Honokiol	Magnolia grandiflora (tea)
56	Biochanin A (4-Methylgenistein	Soy
57	Icariin	Epimedium grandiflorum (Horny goat weed)
58	Indole-3-carbinol	Broccoli
59	Kaempferol	Broccoli
60	Gastrodin (Gastrodine)	Gastrodia elata
61	Kinetin	Coconut
62	Hordenine	Barley
63	Limonin	Orange/Lemons
64	Luteolin	Celery
65	Indirubin	Woad
66	L-carnitine	Beef
67	Methyl-Hesperidin	Citrus fruits
68	Naringin Dihydrochalcone	Grapefruit
69	Polydatin (Piceid)	Grapes
70	Myricetin	Grapes
71	Myricitrin	Myrica cerifera (Bayberry tree)
72	Quercetin (Sophoretin)	Black elder berry
73	Sesamin (Fagorol)	Sesame seeds
74	Naringin	Grapefruits
75	Sorbitol (Glucitol)	Apples
76	Neohesperidin dihydrochalcone	Citrus fruits
77	Rheochrysidin	Rumex crispus
78	Nobiletin	Orange
79	Oleanolic acid	Garlic
80	Salidroside	Rhodiola rosea (tea)
81	Orotic acid	Carrots
82	Osthole	Shishiudo
83	Palmatine chloride	Phellodendron amurense (Amur cork tree)
84	Coenzyme Q10	Red meat
85	Paeonol	Peony
86	Parthenolide	Feverfew
87	Phloretin	Apple
88	Dihydromyricetin	Ampelopsis grossdentata
89	Rhein	Rhubarb
90	Puerarin	Kudzu roots
91	Quercetin dihydrate	Black elder berry
92	Isoliquiritigenin	Liquorice
93	Rutin	Asparagus
94	Salicin	Willow bark
95	Chrysophanic acid	Rhubarb
96	Curcumol	Tumeric

Flow diagram to summarise the experimental process.

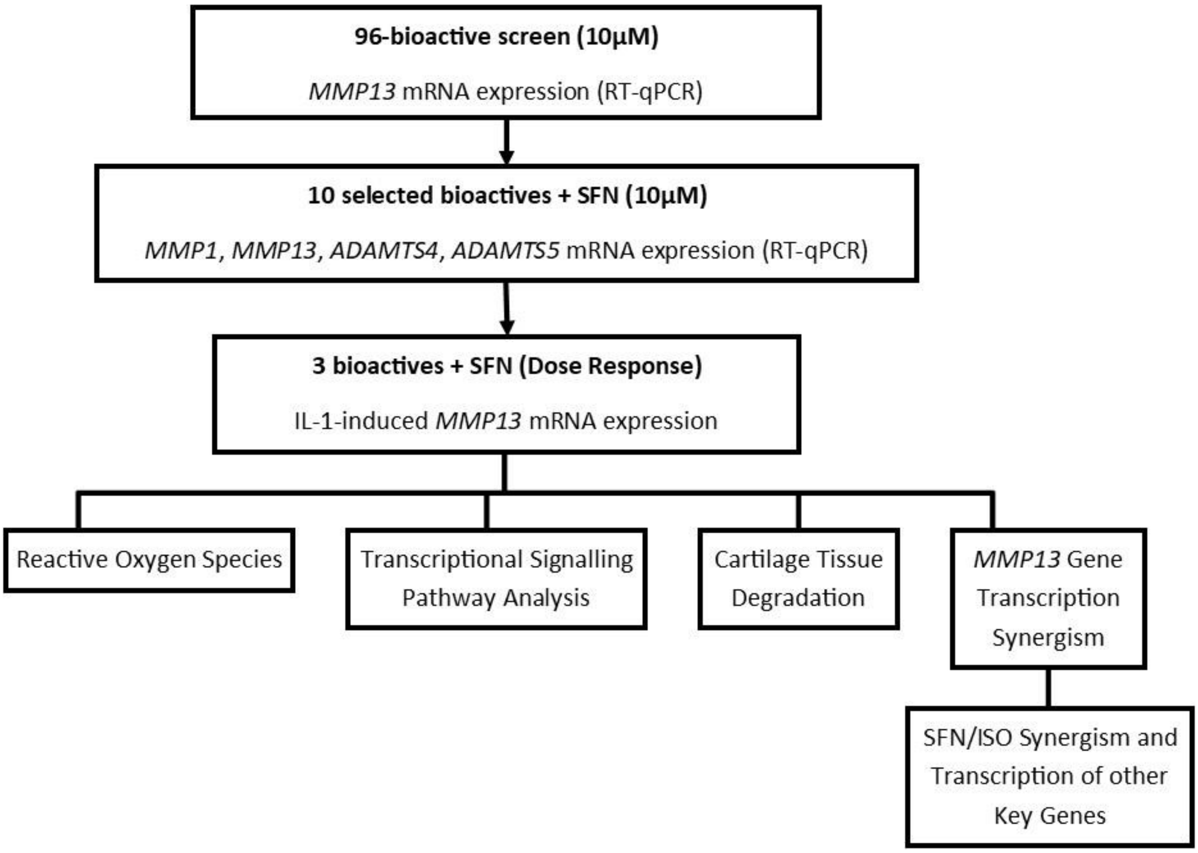

Supplementary Data

***MMP13* Gene Expression**

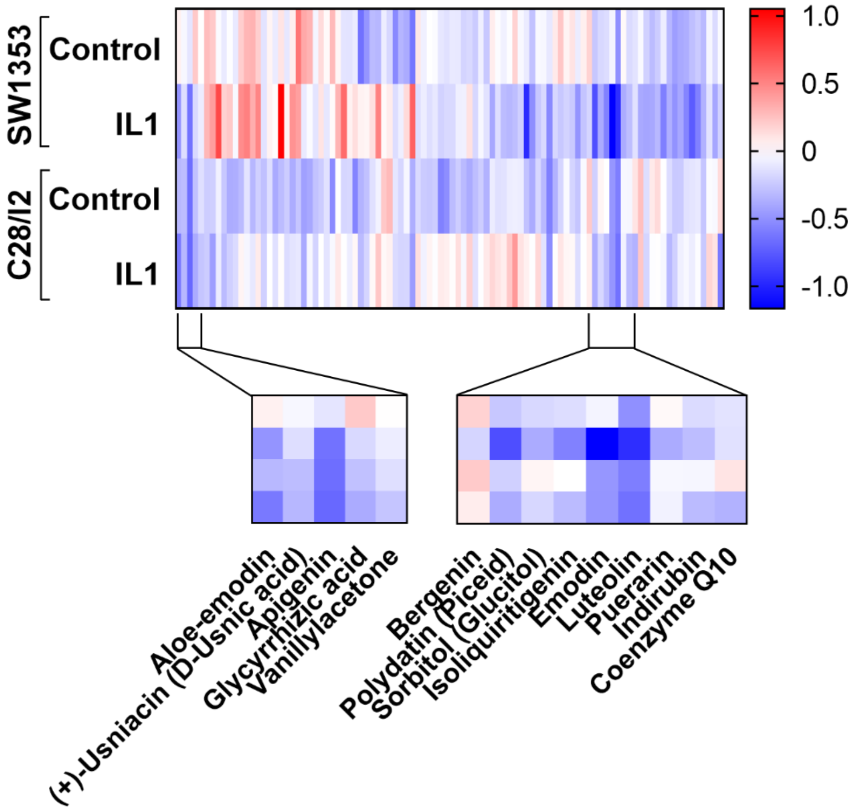

Supplementary S1. Screening bioactives for inhibition of *MMP13* expression. Ninety six bioactives were screened in SW1353 and C28/I2 cell lines for inhibition of *MMP13* expression using RT-qPCR. A minimum of three replicates are used, *n*=1 for each cell line. *MMP13* gene expression was normalised to *18S*. Heat map shows hierarchical clustering of relative expression. Clades containing apigenin, isoliquiritigenin and luteolin are highlighted. Red – increased expression, blue – decreased expression, white – no change, relative to control or IL-1 (10µM) treatment for 6 hours.

Kinase Signalling

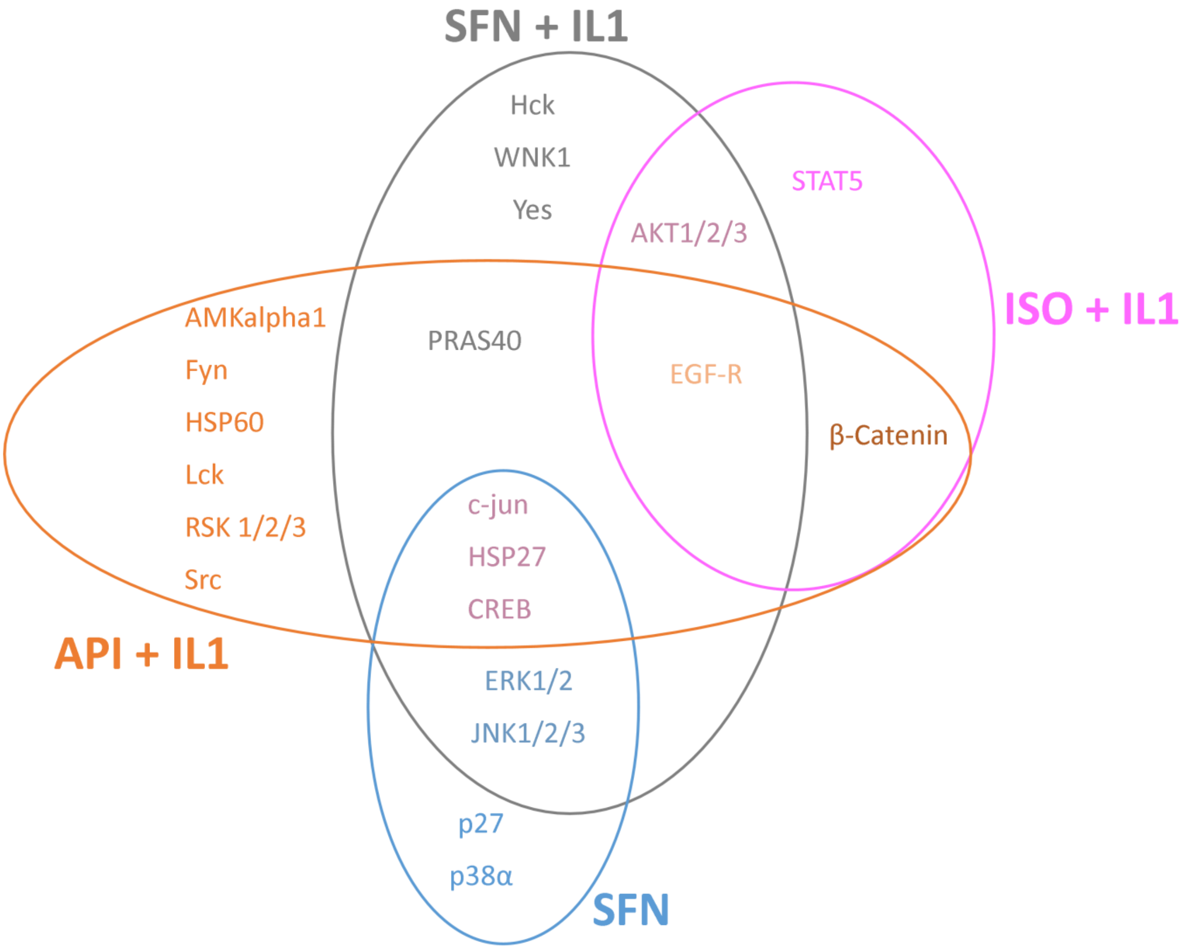

Supplementary S2. **Bioactive regulation of kinase signalling in response to IL-1stimulation in HACs.** HACs were pre-treated with SFN, API or ISO for 30 mins at 10 µM, +/- IL-1 (5ng/ml, 10mins) *n*=1. Phosphorylation was detected using the Proteome Profiler Array (R & D Systems). Semi quantitative analysis of phosphorylation level was measured using ImageJ software and a 1.3 fold cut off point.

Two-Bioactive Combinations

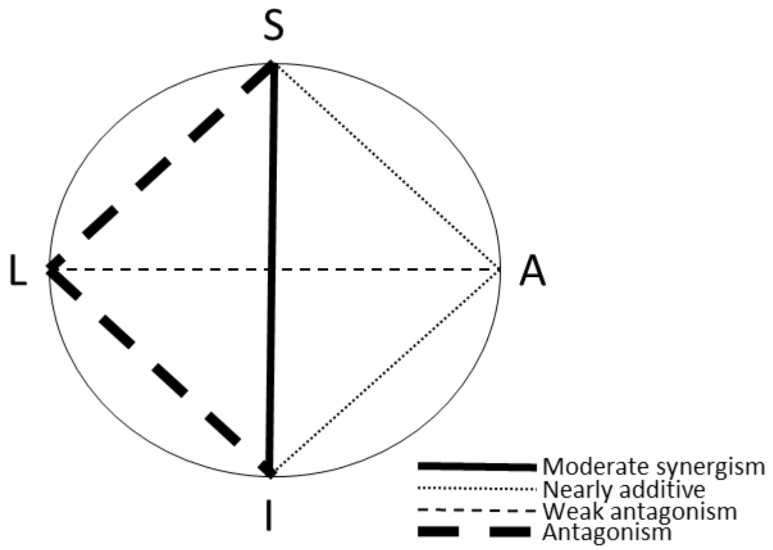

Supplementary S3. Polygonogram summarising the effects of two-bioactive combinations on IL-1-induced *MMP13* gene expression in HACs (*n*=3) by RT-qPCR. Values given are from three independent experiments and each experiment was performed in duplicate. HACs were pre-treated with bioactives in equipotent combination ratios of: S:A:I:L = 0.75:1:0.625:1.25 µM, for 30 mins followed by IL-1 treatment at 5ng/ml for 6 hours. S – sulforaphane, A – apigenin, I – isoliquiritigenin, L – luteolin.

Cytotoxicity

Bioactive	mean	95% CI	summary	adjusted p-value
Control vs. IL-1	5.84	-9.69 to 12.79	ns	0.9994
Control vs. (-)-Hupersine A	8.47	-12.31 to 10.17	ns	0.9996
Control vs. (+)-Usniacin (D-Usnic acid)	9.41	-13.26 to 9.22	ns	0.9993
Control vs. 3-Indolebutyric acid (IBA)	8.38	-12.23 to 10.25	ns	0.9997
Control vs. 4-Methylumbeliferone (4-MU)	9.13	-12.98 to 9.50	ns	0.9994
Control vs. Aesculin	9.91	-13.76 to 8.72	ns	0.999
Control vs. Aloe-emodin	7.16	-11.01 to 11.47	ns	0.9999
Control vs. Aloin (Barbaloin)	4.96	-8.81 to 13.67	ns	0.9991
Control vs. Ammonium Glycyrrhizinate	3.74	-7.59 to 14.9	ns	0.9984
Control vs. Apigenin	10.07	-13.92 to 8.56	ns	0.999
Control vs. Arbutin	8.72	-12.56 to 9.92	ns	0.9996
Control vs. Artemether	10.49	-14.34 to 8.14	ns	0.9987
Control vs. Artesunate	9.45	-13.29 to 9.19	ns	0.9993
Control vs. Asiatic acid	9.93	-13.77 to 8.71	ns	0.999
Control vs. Baicalein	19.03	-22.88 to -0.40	*	0.0354
Control vs. Berberine Hydrochloride	14.03	-17.88 to 4.60	ns	0.7748
Control vs. Bergenin	14.99	-18.84 to 3.64	ns	0.5376
Control vs. Bilobalide	16.63	-20.48 to 2.00	ns	0.22
Control vs. Biochanin A	8.91	-12.76 to 9.72	ns	0.9994
Control vs. B-sitosterol	12.87	-16.72 to 5.76	ns	0.9655
Control vs. Caffeic acid	16.22	-20.07 to 2.41	ns	0.2832
Control vs. Capsaicin	8.77	-12.62 to 9.86	ns	0.9995
Control vs. Chlorogenic acid	16.97	-20.82 to 1.66	ns	0.1761
Control vs. Chrysin	19.1	-22.95 to -0.47	*	0.0333
Control vs. Chrysophanic acid	7.35	-11.19 to 11.29	ns	0.9999
Control vs. Coenzyme Q10	5.73	-9.58 to 12.9	ns	0.9994
Control vs. Curcumol	9.31	-13.15 to 9.33	ns	0.9993
Control vs. Cytisine	6.06	-9.91 to 12.57	ns	0.9996
Control vs. Daidzin	2.50	-6.35 to 16.13	ns	0.9825
Control vs. Dihydroartemisinin	8.30	-12.15 to 10.33	ns	0.9997
Control vs. Dihydromyricetin	6.56	-10.41 to 12.07	ns	0.9997
Control vs. Diosgenin	7.25	-11.1 to 11.39	ns	0.9999
Control vs. Diosmin	2.90	-6.75 to 15.73	ns	0.9916
Control vs. DL-Carnitine hydrochloride	10.94	-14.78 to 7.696	ns	0.9985
Control vs. Ecdysone	8.56	-12.41 to 10.07	ns	0.9996
Control vs. Emodin	8.83	-12.68 to 9.80	ns	0.9995
Control vs. Enoxolone	6.06	-9.91 to 12.57	ns	0.9996
Control vs. Ergosterol	11.07	-14.92 to 7.56	ns	0.9983
Control vs. Fisetin	2.95	-6.80 to 15.69	ns	0.9918
Control vs. Formononetin	4.38	-8.23 to 14.25	ns	0.9988
Control vs. Fumalic acid	4.38	-8.23 to 14.25	ns	0.9988
Control vs. Gastrodin	11.01	-14.85 to 7.63	ns	0.9984
Control vs. Genistin	3.38	-7.23 to 15.26	ns	0.9982

Bioactive	mean	95% CI	summary	adjusted p-value
Control vs. Glycyrrhizic acid	3.74	-7.59 to 14.9	ns	0.9984
Control vs. Gramine	3.95	-7.8 to 14.68	ns	0.9985
Control vs. Gynostemma Extract	4.45	-8.30 to 14.18	ns	0.9988
Control vs. Hesperedin	4.89	-8.73 to 13.75	ns	0.999
Control vs. Hesperetin	5.03	-8.88 to 13.6	ns	0.9991
Control vs. Honokoil	5.68	-9.52 to 12.96	ns	0.9994
Control vs. Hordenine	6.98	-10.83 to 11.65	ns	0.9999
Control vs. Hydroxytryptophan	4.81	-8.66 to 13.82	ns	0.999
Control vs. Icariin	5.40	-9.25 to 13.23	ns	0.9993
Control vs. Indole-3-carbinol	6.35	-10.2 to 12.28	ns	0.9996
Control vs. Indurubin	15.56	-19.40 to 3.08	ns	0.4092
Control vs. Isoliquiritigenin	8.42	-12.27 to 10.21	ns	0.9996
Control vs. Kaempferol	8.47	-12.31 to 10.17	ns	0.9996
Control vs. Kinetin	9.21	-13.06 to 9.43	ns	0.9993
Control vs. L-Carnitine	6.67	-10.52 to 11.97	ns	0.9997
Control vs. Limonen	7.62	-11.47 to 11.01	ns	0.9999
Control vs. Luteolin	6.67	-10.52 to 11.97	ns	0.9997
Control vs. Methyl-Hesperidin	6.67	-10.52 to 11.97	ns	0.9997
Control vs. Myricetin	8.57	-12.42 to 10.06	ns	0.9996
Control vs. Myricitrin	2.39	-6.24 to 16.25	ns	0.9814
Control vs. Naringin	5.97	-9.82 to 12.67	ns	0.9995
Control vs. Naringin Dihydrochalcone	7.94	-11.79 to 10.7	ns	0.9998
Control vs. Neohesperidin dihydrochalcone	3.58	-7.43 to 15.05	ns	0.9983
Control vs. Nobiletin	3.58	-7.43 to 15.05	ns	0.9983
Control vs. Oleanolic acid	4.54	-8.38 to 14.1	ns	0.9988
Control vs. Orotic acid	2.71	-6.55 to 15.93	ns	0.9909
Control vs. Osthole	2.71	-6.55 to 15.93	ns	0.9909
Control vs. Paeonol	10.38	-14.23 to 8.25	ns	0.9988
Control vs. Palmatine chloride	2.63	-6.48 to 16.01	ns	0.9836
Control vs. Parthenolide	4.60	-8.45 to 14.03	ns	0.9989
Control vs. Phloretin	8.13	-11.98 to 10.5	ns	0.9997
Control vs. Polydatin	9.84	-13.69 to 8.79	ns	0.9991
Control vs. Puerarin	5.98	-9.82 to 12.66	ns	0.9995
Control vs. Quercetin (Sophoretin)	9.79	-13.63 to 8.85	ns	0.9991
Control vs. Quercetin dihydrate	7.64	-11.49 to 10.99	ns	0.9999
Control vs. Rhein	20.96	-24.81 to -2.33	**	0.0053
Control vs. Rheochrysidin	6.21	-10.05 to 12.43	ns	0.9996
Control vs. Rutin	6.56	-10.41 to 12.07	ns	0.9997
Control vs. Salicin	7.64	-11.49 to 10.99	ns	0.9999
Control vs. Salidroside	4.54	-8.38 to 14.1	ns	0.9988
Control vs. Sclareol	6.91	-10.76 to 11.72	ns	0.9999
Control vs. Sclareolide	6.66	-10.51 to 11.97	ns	0.9997
Control vs. Sesamin (Fagorol)	4.06	-7.91 to 14.57	ns	0.9986
Control vs. SFN	1.93	-5.78 to 16.7	ns	0.9666

Bioactive	mean	95% CI	summary	adjusted p-value
Control vs. Silibinin	9.05	-12.9 to 9.58	ns	0.9994
Control vs. Silymarin	10.54	-14.39 to 8.09	ns	0.9987
Control vs. Sinomenine	13.55	-17.4 to 5.08	ns	0.8776
Control vs. Sorbitol (Glucitol)	2.86	-6.71 to 15.77	ns	0.9914
Control vs. Stigmasterol	13.62	-17.47 to 5.01	ns	0.8644
Control vs. Tangeretin	7.46	-11.31 to 11.17	ns	0.9999
Control vs. Theobromine	4.00	-7.85 to 14.64	ns	0.9985
Control vs. Troxerutin	8.43	-12.28 to 10.2	ns	0.9996
Control vs. Ursolic acid	7.51	-11.36 to 11.12	ns	0.9999
Control vs. Vanillylacetone	5.80	-9.65 to 12.83	ns	0.9994

Supplementary S4. Cytotoxicity in HACs treated with bioactives. HACs were treated with 10 µM of each bioactive for 24 hours. Lactate dehydrogenase (LDH) was measured using the CytoTox 96® NonRadioactive Cytotoxicity Assay (Promega). Total LDH was measured and data is presented as percent of total cellular LDH (% cytotoxicity). Mean LDH (95% CI) values are shown and adjusted p-values (one way ANOVA with Dunnett's post-test (*n*=3). Values given are from three independent experiments and each experiment was performed in triplicate
